# Integrating miRNA profiling and machine learning for improved thyroid cancer diagnosis

**DOI:** 10.3389/fonc.2026.1838366

**Published:** 2026-09-07

**Authors:** Jia-Ying Xu, Xiao-Ling Zhu, Li Hou, Jun Jiang

**Affiliations:** 1 Department of General Surgery/Department of Thyroid Surgery, The Affiliated Hospital of Southwest Medical University, Luzhou, Sichuan, China; 2 Department of Intensive Care Unit, Deyang People’s Hospital, Deyang, Sichuan, China

**Keywords:** biomarkers, fine needle aspiration, machine learning, miRNA, thyroid cancer

## Abstract

**Background:**

The incidence of thyroid cancer is rising significantly worldwide. It is particularly important to improve the diagnostic accuracy of thyroid cancer. This study aims to integrate miRNAs from fine needle aspiration (FNA) samples of thyroid nodules with machine learning algorithms to improve the accuracy of thyroid cancer diagnosis.

**Methods:**

Differentially expressed miRNA profiles between thyroid cancer and benign thyroid tissues were obtained from the GEO (Gene Expression Omnibus) database (Series GSE116196). Following data preprocessing, least absolute shrinkage and selection operator (LASSO) regression was applied to identify the miRNAs that contributed most to thyroid cancer classification. Subsequently, advanced machine learning algorithms such as logistic regression (GLM), random forest (RF), support vector machine (SVM), XGBoost (XGB), and K-nearest neighbors (KNN) were employed to construct diagnostic models on the training set. Model performance was evaluated on the testing set by receiver operating characteristic (ROC) curve analysis, and the model with the highest area under the curve (AUC) was selected as the top-performing model. Finally, the diagnostic performance of the selected model was independently validated using an external dataset.

**Results:**

Significantly upregulated and downregulated miRNAs in thyroid cancer were identified through DESeq2 analysis. LASSO regression was applied to select the miRNAs with the optimal discriminatory contribution to thyroid nodule characterization, yielding a panel of 13 candidate miRNAs, including hsa-miR-222 (1), hsa-miR-221 (1), and hsa-miR-424 (1). These 13 miRNAs were subsequently used to build disease prediction models using machine learning algorithms. By plotting ROC curves for these machine learning algorithm models and calculating their AUC values, the SVM demonstrated the best performance, with an AUC of 0.788 (95%CI: 0.698-0.873). External validation of the SVM model achieved an accuracy of 80.0%.

**Conclusion:**

This novel integration of miRNA from fine needle aspiration of thyroid nodules and machine learning algorithm holds considerable promise for the clinical translation of miRNA-based diagnostics, enhancing diagnostic precision for indeterminate thyroid nodules.

## Introduction

Thyroid cancer is the most common malignant tumor of the endocrine system. According to recent studies, the incidence of thyroid cancer has significantly increased worldwide (1–3). With over 821,000 cases worldwide in 2022, thyroid cancer ranks as the seventh most common cancer in terms of incidence overall and fifth in women. The incidence rate is three times higher in women than in men (3). The most common type of thyroid cancer is differentiated thyroid cancer (DTC), which includes papillary thyroid carcinoma (PTC) and follicular thyroid carcinoma (FTC). Treatment methods for DTC include surgery, Thyroid Stimulating Hormone (TSH)-suppressive therapy with levothyroxine, and radioactive iodine (4), with surgery being the primary treatment method. Preoperative clinical decision-making hinges critically on distinguishing benign from malignant thyroid nodules. For nodules of indeterminate nature, ultrasound-guided fine needle aspiration biopsy (FNAB) is the preferred procedure for evaluation. The Bethesda system for reporting thyroid cytopathology provides an implied risk of malignancy (ROM) within each indeterminate category (5). The ROM is 45.5-74.1% for atypia of undetermined significance (AUS) and 25-50% for follicular neoplasm (FN). For patients with indeterminate FNAB results (Bethesda III or IV), management options include active surveillance, repeat biopsy, diagnostic surgery, or molecular testing (5).

MicroRNAs (miRNAs) are endogenous small non-coding RNA molecules, typically composed of about 20–22 nucleotides, and play a key role in disease diagnosis, particularly in cancer research (6–8). MiRNAs primarily regulate gene expression by binding to the 3’ untranslated region (UTR) of target mRNAs, thereby promoting mRNA degradation, negatively regulating gene expression, inhibiting protein translation, or facilitating mRNA degradation, thereby affecting key cellular processes associated with cancer development and progression, including cell metabolism, proliferation, differentiation, and apoptosis (8, 9). The relationship between miRNAs and thyroid cancer has been extensively investigated. MiR-221 and miR-222 are typical and well-studied examples of functional miRNAs in thyroid cancer (10). Accumulating evidence indicates that miR-221 and miR-222 are critically involved in the initiation and progression of PTC (11), and miR-222 along with its key target genes may serve as promising biomarkers for thyroid cancer prognosis (12), potentially functioning as important regulators of thyroid cancer immune infiltration (13). Additionally, miR-101 has been shown to inhibit PTC cell proliferation, invasion, and migration while promoting apoptosis by directly targeting CXCL12 (14). Nevertheless, these studies are based on the assumption of a simple linear relationship between a single miRNA and disease; however, biological systems constitute complex networks in which multiple miRNAs may engage in synergistic or antagonistic interactions. Machine learning, a subfield of artificial intelligence, can automatically capture these higher-order interactions, process high-dimensional genomic data, and identify non-linear relationships that conventional statistical methods may overlook, thereby enabling robust predictive modeling (15–17). These computational approaches have been increasingly applied to cancer detection and therapeutic optimization (18), with algorithms such as support vector machines (SVM), k-nearest neighbors (kNN), naïve Bayes (NB), random forests (RF) demonstrating particular utility in oncological research (19, 20).

In this study, we developed a multi-miRNA–based diagnostic model using machine learning methodologies, rather than relying on a single miRNA biomarker. This study extracted a dataset from the GEO (Gene Expression Omnibus) database (Series GSE116196), which was generated by Next-Generation Sequencing (NGS) from FNA eluates of patients’ thyroid nodules. Differentially expressed miRNAs between thyroid cancer and benign thyroid tissues were identified, and a panel of miRNAs that contribute most to the classification of thyroid cancer were screened through Least Absolute Shrinkage and Selection Operator (LASSO) regression. The relative importance of each miRNA in classification was further quantified using feature importance metrics derived from the Random Forest model, which provides a ranking of the most influential variables. These discriminative miRNAs were subsequently used to build disease prediction models using machine learning algorithm in training set, such as logistic regression (GLM), random forest (RF), support vector machine (SVM), XGBoost (XGB), and K-nearest neighbors (KNN). We identified the top-performing model in testing set. Finally, the diagnostic performance of the selected model was independently validated using an external dataset.

In this study, LASSO screened out a panel of 13 miRNAs, among which hsa-miR-222 (1) and hsa-miR-221 (1) ranked at the top, which is consistent with previous studies. By plotting receiver operating characteristic (ROC) curves for these machine learning algorithm models and calculating their area under the curve (AUC) values, the SVM showed the best relative performance, with an AUC of 0.788 (95%CI: 0.698-0.873). External validation confirmed the robustness of the SVM model, achieving an accuracy of 80.0%. This study integrates machine learning methods with traditional biomarker discovery, finding a dynamic, nonlinear decision boundary in high-dimensional space, addressing the limitations of one-size-fits-all solutions in traditional diagnostic methods. This strategy enables more precise judgments based on the overall molecular profile of individual patient (21), and more accurate handling of thyroid nodules with uncertain cytology results (Bethesda III/IV), thereby reducing both unnecessary surgical interventions and missed malignancies. The integrated strategy represents an innovative application of traditional molecular biology techniques and artificial intelligence, enhancing diagnostic accuracy in thyroid cancer diagnosis and promoting biomarker discovery for clinical application.

## Materials and methods

### Data acquisition and preprocessing

For this analysis, the raw miRNA count data were retrieved from the GEO database (Series GSE116196) and processed accordingly. The miRNA expression profiles of these samples were generated using NGS technology, as detailed in the original study (22). A total of 274 samples were obtained from 102 patients, with 71% (n=195) classified as benign and 29% (n=79) as malignant. The expression levels of miRNAs were quantified and normalized using multiplexing technologies.

To ensure the appropriate matching of expression data and clinical information, both datasets were aligned based on patient sample identifiers. The clinical dataset provided information on the patient’s diagnosis, categorized as either “Normal” (benign) or “Tumor” (malignant), which was used as the outcome variable in subsequent analyses.

### Differential expression analysis

Differentially expressed miRNAs between benign and malignant thyroid samples were identified using DESeq2 (version 1.30.1), a widely-used tool for RNA-Seq analysis. Raw count data were input into the DESeq2 pipeline, where differential expression was assessed by comparing “Normal” and “Tumor” groups. For each miRNA, the significance of the differential expression was evaluated using the Wald test. We applied a threshold of adjusted p-value (padj) < 0.05 and a fold-change threshold of |log_2_FC| > 1 to select significantly differentially expressed miRNAs (23–26). These thresholds were selected to balance statistical stringency and biological relevance, minimizing false positives while capturing miRNAs with substantial expression changes that are more likely to have functional consequences.

### Identification of key miRNAs using LASSO

To refine the list of differentially expressed miRNAs, we employed a LASSO regression model, which is particularly suitable for high-dimensional data such as miRNA expression profiles. The LASSO method was used to perform feature selection, identifying a set of miRNAs that contribute most strongly to the classification of “Tumor” vs “Normal” status. The LASSO model was trained using 10-fold cross-validation to ensure robustness and avoid overfitting. The optimal regularization parameter (lambda) was selected based on the minimum cross-validation error. The miRNAs with non-zero coefficients were selected as the final feature set for downstream modeling. The importance of each miRNA in classification was determined using feature importance metrics from the Random Forest model, which provides a ranking of the most influential variables.

### Model building and evaluation

Various machine learning models were constructed to predict thyroid nodule malignancy based on the selected miRNA features. These models included GLM, RF, SVM, XGB, and KNN. For each model, a 5-fold cross-validation approach was used to evaluate performance on the training set, optimizing for the AUC-ROC as the performance metric. The dataset was randomly partitioned into a training set (70%) and an independent testing set (30%) using a fixed random seed to ensure reproducibility (27–29). The 70/30 split was chosen to provide sufficient data for model training while retaining an adequately sized hold-out set for unbiased performance evaluation. This allocation is widely adopted in biomedical machine learning studies and represents a practical balance between maximizing training data and ensuring reliable external validation.

Model performance was evaluated using AUC, accuracy, sensitivity, specificity, and positive/negative predictive values. These metrics were computed based on predictions made on the testing set, where each model’s probability scores were used to assess the probability of malignancy. The predictive performance of each model was quantified by the area under the ROC curve (AUC). Statistical comparisons between models were performed using resampling techniques (e.g., resampling and ROC analysis), and the model with the highest AUC was considered the best performing.

### External validation

To validate the predictive performance of the miRNA-based signature, we used an independent external validation cohort consisting of 30 additional thyroid nodule samples. These samples were classified as either benign or malignant based on histopathological examination. This study was approved by the Ethics Committee of the Affiliated Hospital of Southwest Medical University (KY2025756), and all patients signed informed consent. Total RNA was extracted from all tissue specimens for RT-qPCR testing.

The performance of the miRNA signature was assessed by applying the LASSO-selected miRNAs and the best-performing classification model to this external validation set. The accuracy, sensitivity, and specificity of the model were computed to determine its utility in a real-world clinical context.

## Results

### Identification of differentially expressed miRNAs

**Figure 1:**
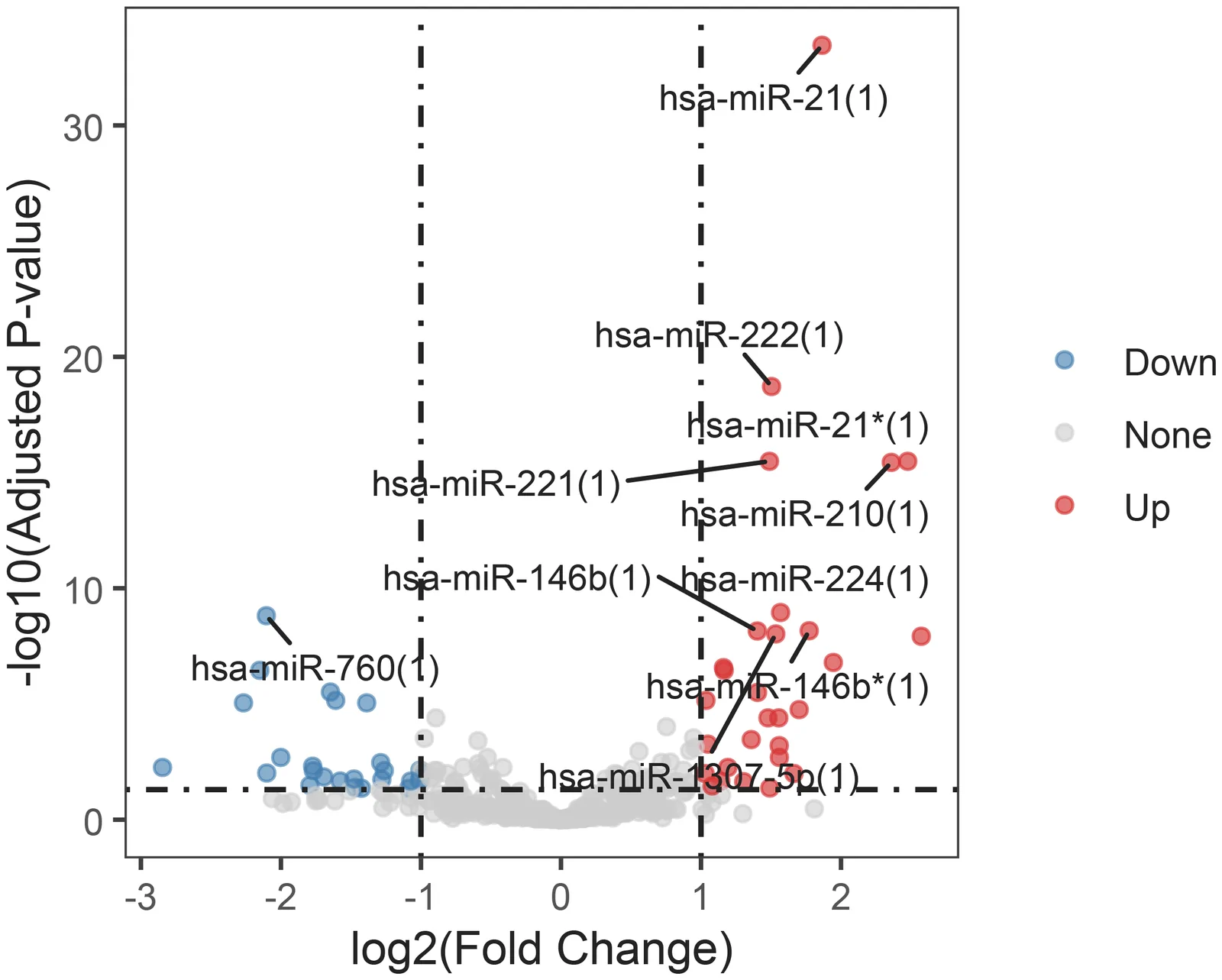
Volcano plot of differential expression miRNAs between thyroid cancer and benign thyroid nodule samples. The differential expression miRNAs are obtained based on criteria of log fold-change (log_2_FC) < -1 for downregulated miRNAs and (log2FC) > 1 for upregulated miRNAs with an adjusted P-value (padj) < 0.05.

**Figure 2:**
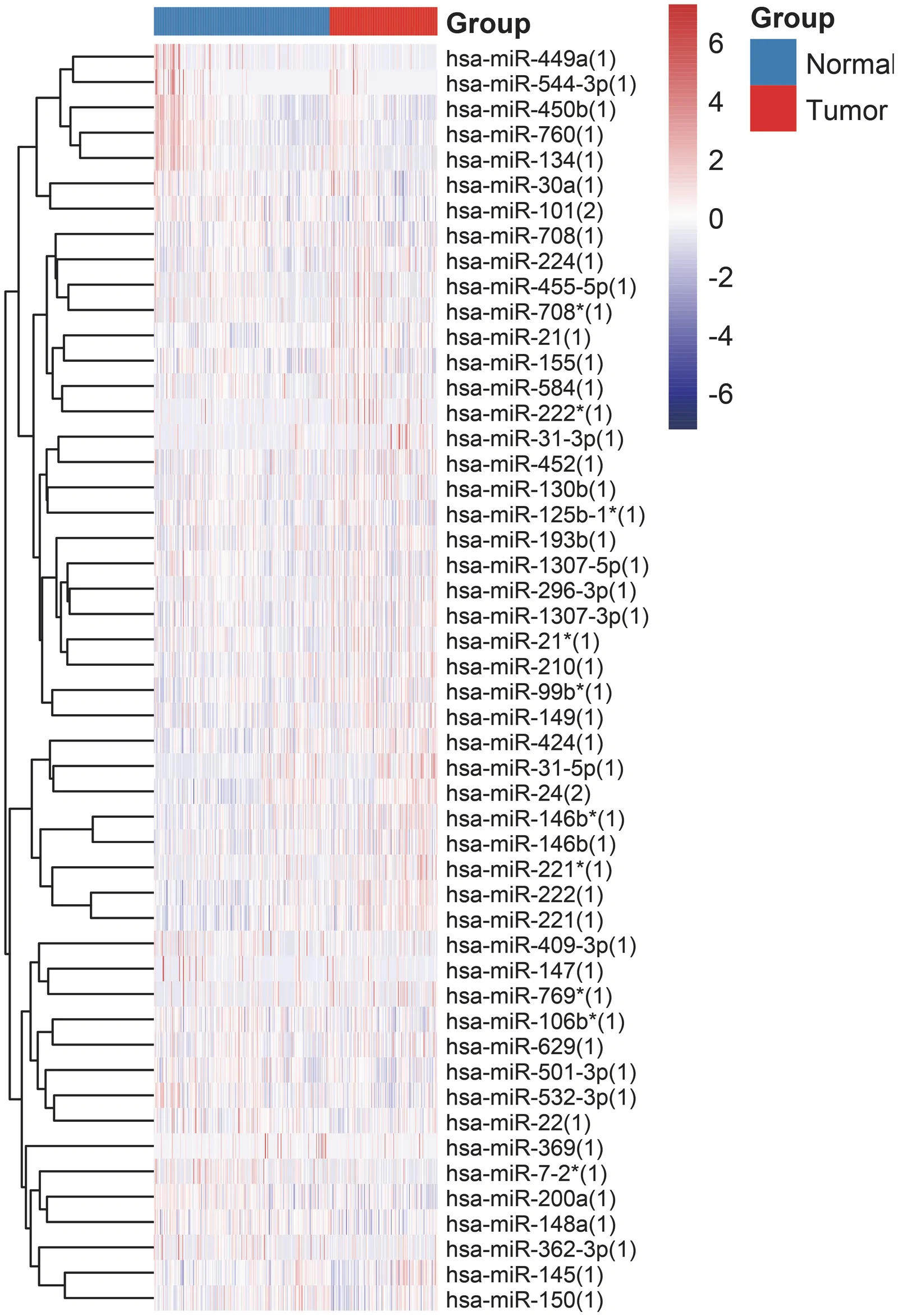
Heatmap illustrating the results of re-analysis of miRNA dataset (series GSE116196). There are 54 differentially expressed miRNAs here, and their names are on the right. The asterisk (*) denotes the passenger ("star") strand of the miRNA duplex, following the miRBase nomenclature used in the source dataset annotation (GSE116196); it does not indicate statistical significance. The numbers in parentheses, e.g., (1), denote distinct genomic loci encoding the same mature miRNA, as annotated in the source dataset.

We performed comprehensive bioinformatics analysis using series GSE116196 from GEO database. The dataset comprised 274 samples from 102 patients, of which 195 (71%) were classified as benign and 79 (29%) as malignant. Differentially expressed miRNAs were screened using an adjusted p-value (padj) < 0.05 and |log_2_FC| > 1 as thresholds, ultimately identifying 54 differentially expressed miRNAs. Among them, 29 miRNAs were upregulated in thyroid cancer tissues, and 25 miRNAs were downregulated. The volcano plot (**Figure 1**) illustrates the differential expression results, with red and blue dots representing significantly upregulated and downregulated miRNAs respectively, gray dots indicating non-significant changes. The horizontal dashed line indicates adjusted P (padj) = 0.05 threshold, the vertical dashed lines represent |log_2_FC| = 1 cutoffs. The top 10 miRNAs with the highest differential expression are labeled with their identifiers. Nine out of ten were upregulated: hsa-miR-21 (1), hsa-miR-222 (1), hsa-miR-21* (1), hsa-miR-221 (1), hsa-miR-210 (1), hsa-miR-224 (1), hsa-miR-146b* (1), hsa-miR-146b (1), and hsa-miR-1307-5p (1), while one miRNA, hsa-miR-760 (1), was downregulated. The heatmap (**Figure 2**) displays the expression profiles of the differentially expressed miRNAs in thyroid cancer tissues and benign thyroid nodule tissues.

### Least absolute shrinkage and selection operator regression

**Figure 3:**
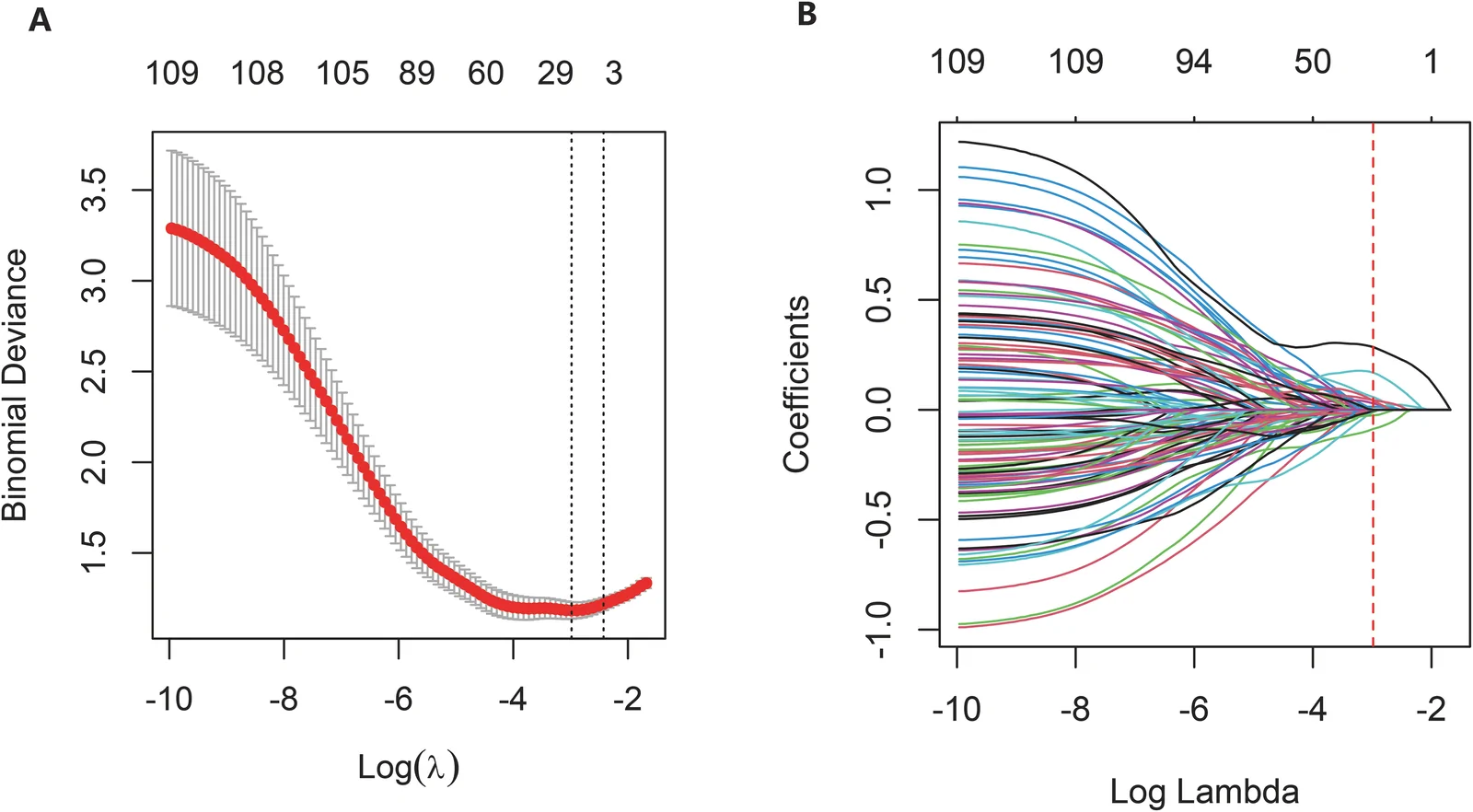
**(A)** Binomial deviance plot showing the performance of the model as a function of the log-transformed λ values. The red line indicates the optimal λ value selected by cross-validation, where the deviance is minimized. **(B)** Lasso regression coefficient plot, showing the change in coefficients of selected variables as the L1 regularization parameter (λ) increases. The coefficients are represented by colored lines, with each line corresponding to a miRNA.

**Figure 4:**
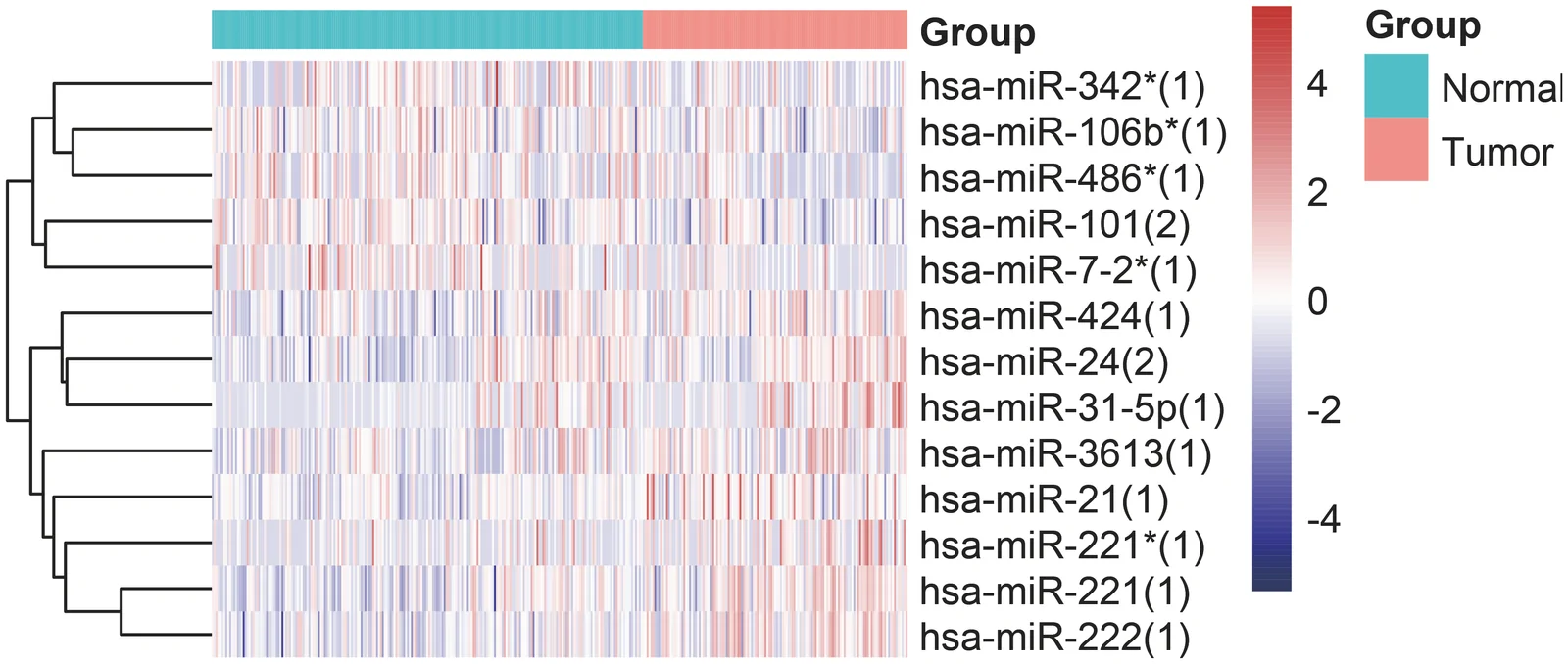
The heatmap shows the expression of miRNAs selected by LASSO in thyroid cancer and benign thyroid nodule samples. Red represents miRNAs that are upregulated in thyroid cancer tissues, while blue represents miRNAs that are downregulated in thyroid cancer tissues. The asterisk (*) denotes the passenger ("star") strand of the miRNA duplex, following the miRBase nomenclature used in the source dataset annotation (GSE116196); it does not indicate statistical significance. The numbers in parentheses, e.g., (1) and (2), denote distinct genomic loci encoding the same mature miRNA, as annotated in the source dataset.

**Figure 5:**
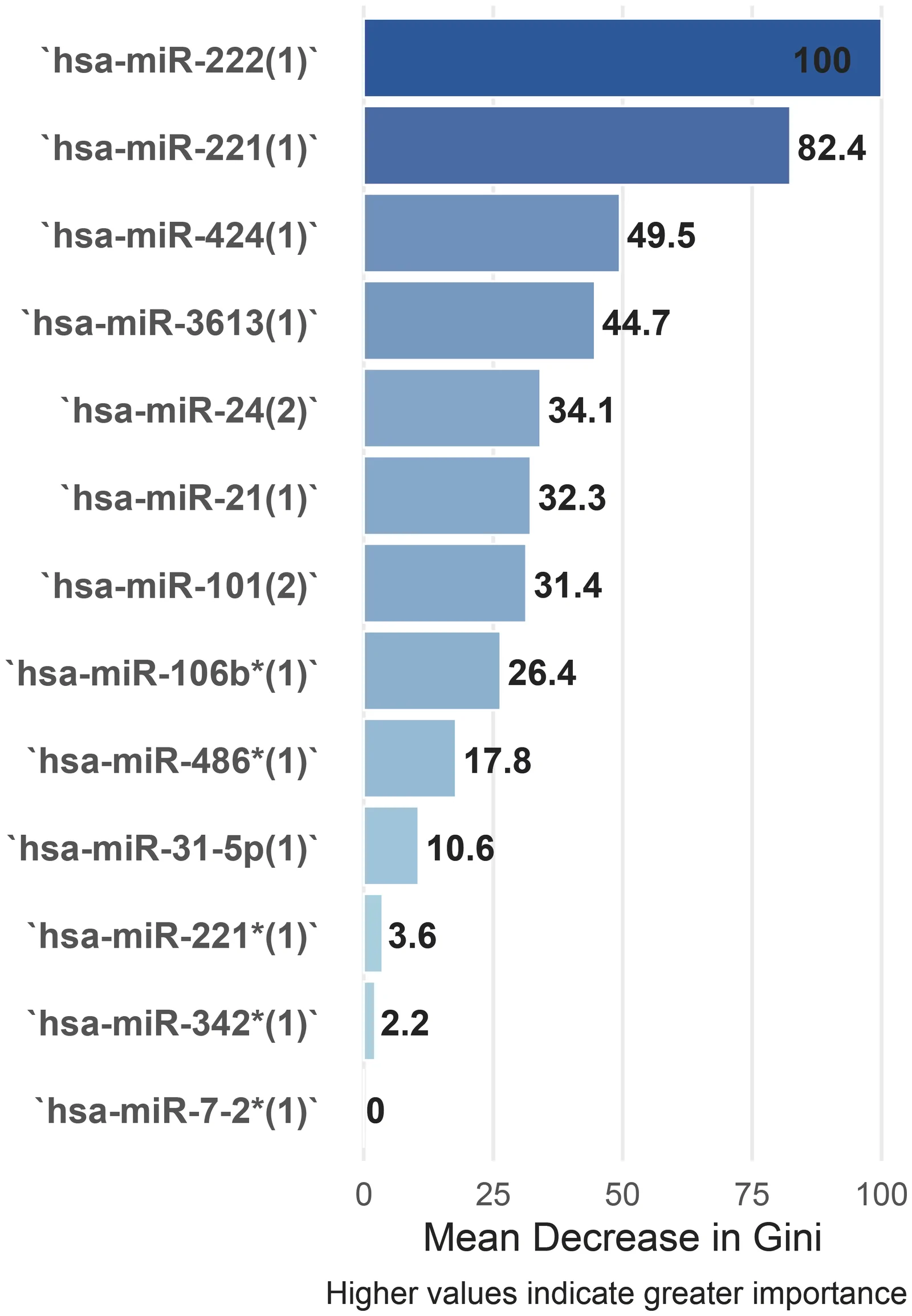
Feature importance ranking of discriminative miRNAs identified by Random Forest algorithm. Horizontal bar chart displays importance scores (x-axis) for each miRNA identifier (y-axis).

Using the LASSO regression analysis (**Figure 3**), a panel of miRNAs that contributed most to distinguishing benign from malignant cases were identified. These discriminative miRNAs were employed in downstream machine learning algorithms to establish predictive models for thyroid cancer. LASSO regression ultimately screened 13 candidate miRNA biomarkers: hsa-miR-342* (1), hsa-miR-106b* (1), hsa-miR-486* (1), hsa-miR-101 (2), hsa-miR-7-2* (1), hsa-miR-424 (1), hsa-miR-24 (2), hsa-miR-31-5p (1), hsa-miR-3613 (1), hsa-miR-21 (1), hsa-miR-221* (1), hsa-miR-221 (1), and hsa-miR-222 (1) (**Figure 4**). Feature importance analysis using the Random Forest algorithm further identified these discriminative miRNAs contributing to cancer classification (**Figure 5**), with hsa-miR-222 (1) emerging as the most influential biomarker with the highest importance score, followed by hsa-miR-221 (1) and hsa-miR-424 (1).

### Model construction and evaluation

**Figure 6:**
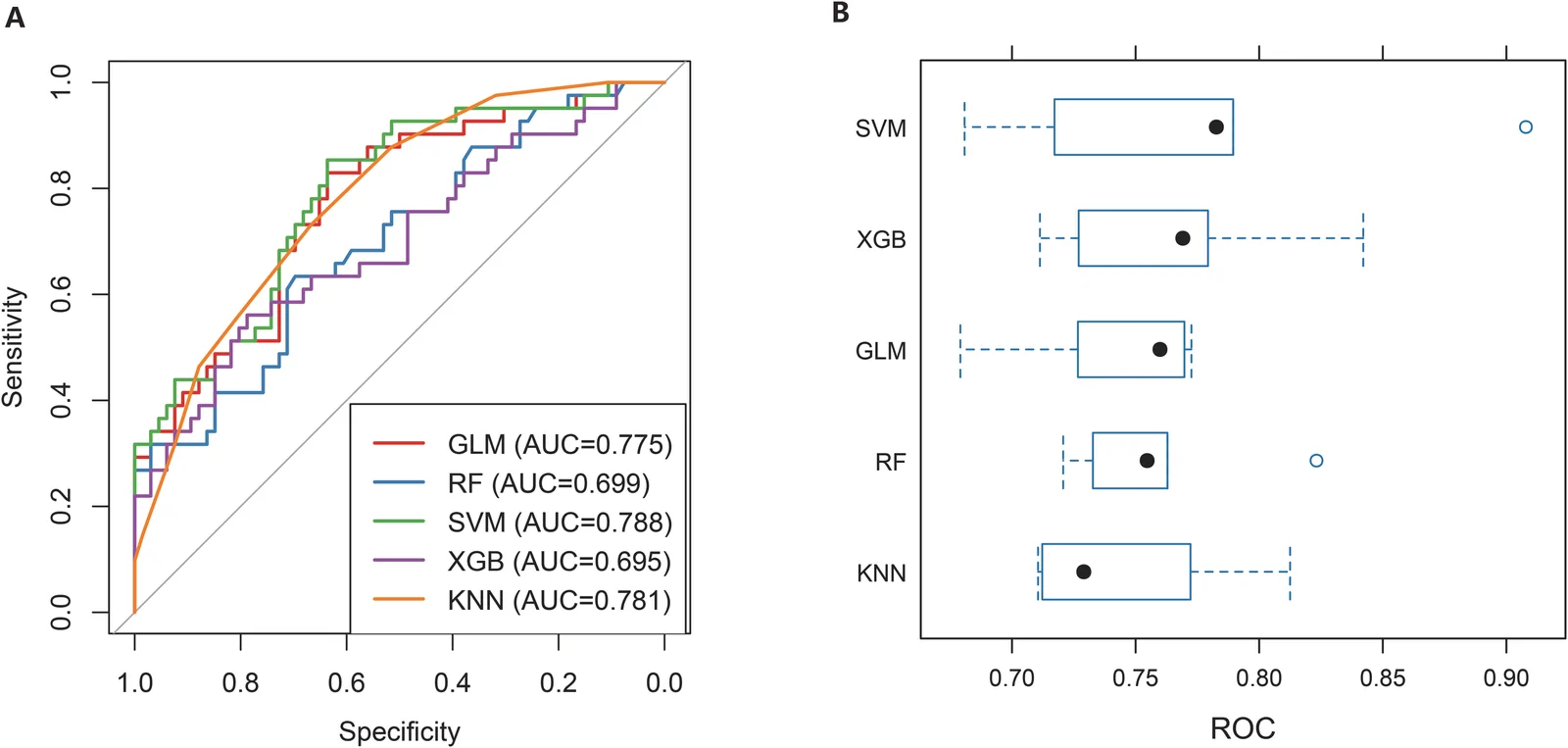
**(A)** ROC curves for the classification performance of various models. Each curve represents the trade-off between sensitivity and 1-specificity for the selected model. **(B)** Visualization of AUC value comparison for various machine learning models.

The dataset was divided into 70% for the training set and 30% for the testing set randomly. These discriminative miRNAs were used as inputs for machine learning algorithms in the training set to predict the malignancy risk of thyroid nodules. These machine learning algorithms included GLM, RF, SVM, XGB, and KNN. Subsequently, we validated the performance of these algorithms in the testing set. By comparing the AUC values of these algorithms, the SVM was identified as the most accurate one (SVM, AUC = 0.788, 95%CI: 0.698-0.873; GLM, AUC = 0.775, 95%CI: 0.682-0.861; KNN, AUC = 0.781, 95%CI: 0.689-0.863; XGB, AUC = 0.695, 95%CI: 0.589-0.798; RF, AUC = 0.699, 95%CI: 0.596-0.799) (**Figure 6**; **Supplementary Table 1**).

### External validation

**Figure 7:**
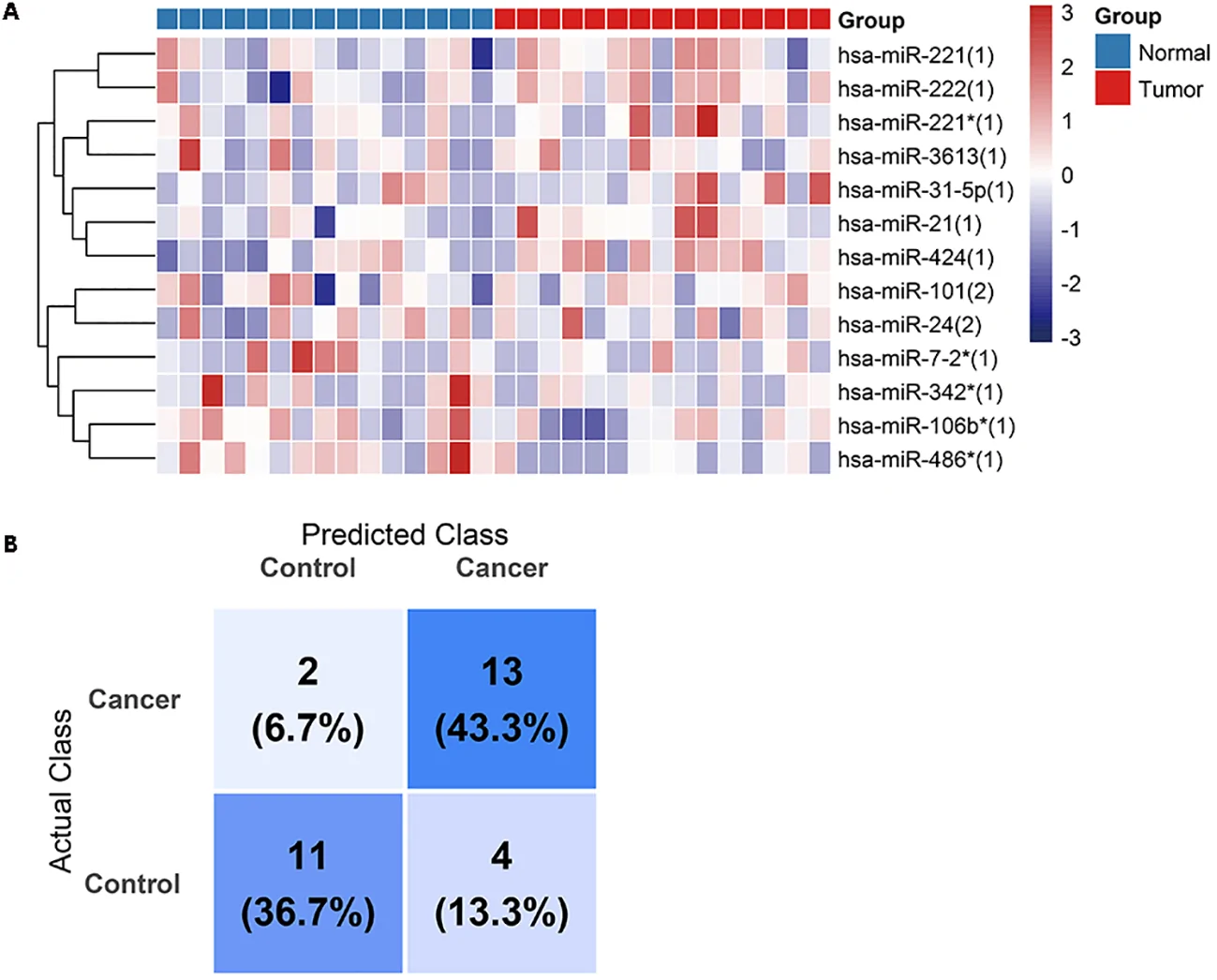
**(A)** This heatmap shows the expression of miRNAs selected by LASSO in independent data. Red represents miRNAs that are upregulated in thyroid cancer tissues, while blue represents miRNAs that are downregulated in thyroid cancer tissues. **(B)** Confusion matrix validation of the SVM model accuracy for thyroid cancer on independent data.

An independent dataset that had not been exposed to the machine learning algorithm validated the performance of the selected algorithm (SVM). We collected 15 cases of thyroid cancer tissue and 15 cases of benign thyroid tissue in the Affiliated Hospital of Southwest Medical University, all of which were confirmed by pathological examination. First, RT-qPCR verified the expression of the discriminative miRNAs (**Figure 7A**). Subsequently, the expression of the discriminative miRNAs was input into the SVM to validate the performance. The confusion matrix (**Figure 7B**) results demonstrated that the SVM model achieved an accuracy of 80.0%, an error rate of 20.0%, a precision of 0.76 and a recall of 0.87, and an F1 score of 0.81.

## Discussion

With the rising incidence of thyroid cancer, the diagnosis remains a critical clinical challenge. The biggest challenge in the current diagnosis and treatment of thyroid nodules is the diagnostic dilemma of Bethesda III/IV (cytologically indeterminate) nodules. Despite evaluation by ultrasound and FNAB, the nature of some thyroid nodules remains inconclusive, which may potentially lead to unnecessary surgery or missed malignancies. Numerous studies have shown that miRNAs can serve as potential biomarkers and therapeutic targets for human diseases (30), and miRNAs can be stably measured in various sample types, making them promising biomarker candidates (31). Research more than a decade ago found that FNA miRNA analysis could serve as a valuable adjunct in the management algorithm of patients with thyroid nodules (32). Compared to other biomarkers such as metabolites and proteins, miRNAs have several distinct advantages. First, unlike low-abundance metabolite and protein biomarkers, low-abundance miRNA biomarkers can be amplified and detected using real-time quantitative polymerase chain reaction (RT-qPCR). Second, miRNA biomarkers are located upstream in regulatory cascades, allowing them to be diagnosed earlier than metabolites or proteins. Finally, novel miRNA biomarkers can be more readily identified through high-throughput methods such as deep sequencing and oligonucleotide microarrays, which offer greater throughput than the mass spectrometry typically employed for proteins and metabolites (33, 34). Recent studies have indicated that the expression levels of specific miRNAs in thyroid tumor tissues correlate with various clinicopathological features, including tumor size, multifocality, capsular invasion, extrathyroidal extension, as well as lymph node and distant metastases. For these reasons, the role of miRNAs has come into play in distinguishing AUS cases as benign or malignant (35).

The dataset for this study was obtained from the GEO database (Series GSE116196). This dataset comprises 274 samples from 102 patients, with miRNA expression profiles derived from the FNA eluent of thyroid nodules. The cells and nucleic acids in FNA eluent originate directly from the thyroid nodule. Compared with circulating miRNAs, which contain substantial quantities of miRNAs derived from blood cells, vascular cells, and other normal tissues, FNA samples exhibit a considerably higher proportion of target cells and an improved signal-to-noise ratio, thereby facilitating the detection of authentic tumor-associated miRNA expression. Furthermore, this procedure seamlessly integrates with existing clinical workflows, as FNA is already a standard procedure in the diagnosis and management of thyroid nodules. Following routine puncture, a small volume of saline is used to flush the needle to collect the eluate, without causing additional trauma to the patient, making sample collection easier to approve both ethically and operationally.

High-throughput methods such as RNA-sequencing (RNA-seq) were employed for miRNA detection in eluate specimens, which can assess the expression of thousands of genes in a single assay, has revolutionized the field of cancer biomarker discovery (36). LASSO identified 13 discriminative miRNAs, with hsa-miR-222 (1) emerging as the most influential biomarker with the highest importance score, followed by hsa-miR-221 (1) and hsa-miR-424 (1). This result is consistent with previous research findings. MiR-221 and miR-222 are typical and well-studied examples of functional miRNAs in thyroid cancer (10). The inclusion of these discriminative miRNAs in the model construction suggests that nucleic acids within the tumor microenvironment may be effectively released and enriched in the residual eluates after FNA puncture. In addition, miR-424 (37), miR-21 (38, 39), and miR-31-5p (40), which were incorporated into the model construction, function as oncogenic factors, whereas miR-101 (14, 41), miR-106b (42), miR-486 (43–45), and miR-7-2 (46) have been proven to be tumor suppressors. Currently, there is no relevant research on miR-342 and miR-3613 in thyroid cancer; however, miR-342 plays a suppressive role in pancreatic cancer (47) and osteosarcoma (48), while miR-3613 exhibits a suppressive activity in breast cancer (49). Therefore, we speculate that miR-342 and miR-3613 may similarly act as tumor suppressors in thyroid cancer.

Although these dysregulated miRNAs hold considerable promise as biomarkers for thyroid cancer, research has largely focused on the association between individual miRNA and the disease. However, individual miRNAs lack adequate specificity. For instance, miRNAs that are highly expressed in thyroid cancer (such as the miR-221/222 family) are not exclusive to the thyroid. They are also associated with other solid tumors [such as liver cancer (50) and breast cancer (51)] as well as non-tumor diseases (such as chronic inflammation) (52). Consequently, reliance on a single miRNA biomarker may lead to false positive issues, and be insufficient for distinguishing thyroid cancer from other diseases. Several miRNAs have demonstrated utility in thyroid cancer classification. Among the miRNAs examined, miRNA-375 has shown effectiveness in distinguishing MTC from other malignant and benign thyroid tumors (53). Similarly, miRNA-484, miRNA-148b-3p, and miRNA-885-5p have been identified as particularly effective in distinguishing FTC (35). Traditional biostatistical methods carry risks of multicollinearity and overfitting when handling high-dimensional miRNA data, and they are poorly equipped to capture synergistic or antagonistic interactions among multiple miRNAs. In this study, 13 discriminative miRNAs identified by LASSO were incorporated into machine learning algorithm inputs to construct prediction models. The machine learning algorithms are capable of capturing complex nonlinear relationships among different variables, which are embedded in the dataset and difficult for conventional approaches to discern. This study employs five-fold cross-validation to ensure model generalizability and to evaluate performance on data independent of the training set, thus avoiding overfitting. Based on the GEO dataset, five prediction models were built to assess the malignancy potential of thyroid nodules. AUC values of each model were compared (ranging from 0.695 to 0.788), with the SVM achieving the highest AUC of 0.788. Therefore, in this study, the SVM model was considered to be the optimal model for predicting thyroid cancer. To validate the predictive performance of the miRNA-based signature, an independent external validation cohort was input, with an accuracy of 80.0%, an error rate of 20.0%, a precision of 0.76 and a recall of 0.87, and an F1 score of 0.81. These results indicate that the model possesses clinical applicability. Clinicians can integrate patients’ demographic and clinical characteristics, such as age, nodule size, ultrasound TI-RADS classification, FNA Bethesda classification, as well as the prediction results of the predictive model established in this study, which can greatly improve the diagnostic accuracy for patients with thyroid nodules classified as Bethesda III/IV.

The results of this study hold considerable prospects for clinical translation. These 13 discriminative miRNAs could potentially be made into a quantitative polymerase chain reaction (qPCR) panel, which can substantially reduce the economic and time costs when detecting miRNAs in the eluate. Based on FNA cytological diagnosis, miRNA testing can be performed using the eluent from the same puncture needle and input into a machine learning model subsequently, providing supplementary molecular information without additional invasive operation. This approach holds the promise of establishing an integrated diagnostic paradigm combining cytology, molecular biology, and artificial intelligence, thereby offering more precise decision support for the clinical management of thyroid nodules.

Existing clinical diagnostic tools, like Afirma (microarray-based) and ThyroSeq (NGS -based), are effective for managing indeterminate thyroid nodules, primarily by ruling out malignancy and reducing unnecessary diagnostic surgeries. Both tools demonstrate excellent sensitivity and negative predictive value (NPV), however, their specificity remains suboptimal (54, 55). Afirma leverages whole-transcriptome profiling (~10,000 genes) and thus possesses a theoretical informational advantage; however, its limited specificity and high cost restrict its accessibility, particularly in China. In contrast, the SVM predictive model we developed utilizes a compact panel of 13 distinctive miRNAs. While this approach may not fully capture the molecular heterogeneity of thyroid cancer, it offers moderate diagnostic performance with the advantages of lower cost, simpler operation, and greater feasibility in primary care hospitals or resource-limited settings. Furthermore, existing commercial tools were predominantly developed and validated in Western populations, which may limit their generalizability to Asian cohorts. Our SVM model, by contrast, was constructed and validated using data from an Asian population, potentially offering improved applicability in this demographic.

Given the inherent diagnostic limitations of single molecular markers in Bethesda III/IV nodules, multimodal fusion strategies have emerged as promising approaches to enhance diagnostic accuracy. For instance, Zhong and colleagues integrated clinical features, ultrasound radiomics, and BRAF V600E, achieving an external validation AUC of 0.824 in Bethesda III nodules (56); Wu and colleagues demonstrated that combining TI-RADS with BRAF V600E provided superior diagnostic performance in Bethesda III-IV nodules compared with any single marker alone (57). Given the moderate diagnostic efficacy of the SVM model developed in this study, it is better positioned as a complementary tool to existing molecular assays rather than a standalone replacement. Therefore, future investigations should aim to integrate this model with clinical variables (e.g., age, sex, and nodule size), ultrasound TI-RADS classification, and molecular markers such as BRAF V600E, employing advanced machine learning and deep learning algorithms to construct a multimodal joint prediction model. Such an approach may further improve diagnostic accuracy for Bethesda III/IV nodules (35).

Our study has several limitations. Although our model can be generalized to meet the needs of real clinical practice, its limitations must still be acknowledged. First, as a single-center study, the sample size is relatively limited. Although independent validation was performed, large-scale multicenter external datasets are still required to verify the generalizability of the model. Second, the ‘black box’ nature of machine learning models may undermine clinicians’ trust in the prediction results. In the future, we intend to use methods such as Shapley Additive exPlanations (SHAP) analysis to improve the interpretability of the model. Third, the collection and processing standards of FNA eluent have not yet been unified, which may be a potential factor affecting the stability of the results. Finally, the selection of appropriate and consistent reference genes is required to ensure accurate and comparable results in RT-qPCR.

## Conclusion

This study integrates traditional bioinformatics with advanced machine learning algorithms. The miRNA machine learning prediction model for thyroid cancer using FNA eluate is positioned in the intermediate zone between tissue biopsy (gold standard but invasive) and liquid biopsy (non-invasive but with weak signals). This approach combines the strength of lesion signals with the convenience of non-invasive operation, substantially improving the accuracy of thyroid cancer diagnosis, and is an ideal tool for Bethesda III/IV nodules. Future research will focus on expanding the sample size for prospective validation and exploring the integration of this model with ultrasound radiomics and cytological features to construct a multimodal diagnostic model, aiming to achieve optimal management of thyroid nodules.

## Data Availability

The datasets analyzed in this study are publicly available. The miRNA sequencing data were obtained from the Gene Expression Omnibus (GEO) repository under accession number GSE116196 (https://www.ncbi.nlm.nih.gov/geo/query/acc.cgi?acc=GSE116196). The RT-qPCR data generated for external validation in this study are available from the corresponding author upon reasonable request.

## References

[1] Han B Zheng R Zeng H Wang S Sun K Chen R . Cancer incidence and mortality in China, 2022. J Natl Cancer Cent. (2024) 4:47–53. doi: 10.1016/j.jncc.2024.01.006 39036382 PMC11256708

[2] Miranda-Filho A Lortet-Tieulent J Bray F Cao B Franceschi S Vaccarella S . Thyroid cancer incidence trends by histology in 25 countries: A population-based study. Lancet Diabetes Endocrinol. (2021) 9:225–34. doi: 10.1016/S2213-8587(21)00027-9 33662333

[3] Bray F Laversanne M Sung H Ferlay J Siegel RL Soerjomataram I . Global cancer statistics 2022: Globocan estimates of incidence and mortality worldwide for 36 cancers in 185 countries. CA Cancer J Clin. (2024) 74:229–63. doi: 10.3322/caac.21834 38572751

[4] Ringel MD Sosa JA Baloch Z Bischoff L Bloom G Brent GA . 2025 American Thyroid Association management guidelines for adult patients with differentiated thyroid cancer. Thyroid Off J Am Thyroid Assoc. (2025) 35:841–985. doi: 10.1177/10507256251363120 40844370 PMC13090833

[5] Ali SZ Baloch ZW Cochand-Priollet B Schmitt FC Vielh P VanderLaan PA . The 2023 Bethesda system for reporting thyroid cytopathology. Thyroid Off J Am Thyroid Assoc. (2023) 33:1039–44. doi: 10.1089/thy.2023.0141 37427847

[6] Nemeth K Bayraktar R Ferracin M Calin GA . Non-coding rnas in disease: From mechanisms to therapeutics. Nat Rev Genet. (2023) 25:211–32. doi: 10.1038/s41576-023-00662-1 37968332

[7] Bhatti GK Khullar N Sidhu IS Navik US Reddy AP Reddy PH . Emerging role of non-coding rna in health and disease. Metab Brain Dis. (2021) 36:1119–34. doi: 10.1007/s11011-021-00739-y 33881724 PMC8058498

[8] Sohel MMH . Circulating micrornas as biomarkers in cancer diagnosis. Life Sci. (2020) 248:117473. doi: 10.1016/j.lfs.2020.117473 32114007

[9] Chen L-L Kim VN . Small and long non-coding rnas: Past, present, and future. Cell. (2024) 187:6451–85. doi: 10.1016/j.cell.2024.10.024 39547208

[10] Hu Y Wang H Chen E Xu Z Chen B Lu G . Candidate micrornas as biomarkers of thyroid carcinoma: A systematic review, meta-analysis, and experimental validation. Cancer Med. (2016) 5:2602–14. doi: 10.1002/cam4.811 27465286 PMC5055193

[11] Ye T Zhong L Ye X Liu J Li L Yi H . Mir-221-3p and mir-222-3p regulate the socs3/stat3 signaling pathway to downregulate the expression of nis and reduce radiosensitivity in thyroid cancer. Exp Ther Med. (2021) 21:652. doi: 10.3892/etm.2021.10084 33968182 PMC8097237

[12] Kondrotienė A Daukša A Pamedytytė D Kazokaitė M Žvirblienė A Daukšienė D . Plasma-derived mirna-222 as a candidate marker for papillary thyroid cancer. Int J Mol Sci. (2020) 21:6445. doi: 10.3390/ijms21176445 32899424 PMC7503340

[13] Zhang T Chen Y Lin W Zheng J Liu Y Zou J . Prognostic and immune-infiltrate significance of mir-222-3p and its target genes in thyroid cancer. Front Genet. (2021) 12:710412. doi: 10.3389/fgene.2021.710412 34737762 PMC8562566

[14] Chen F Yang D Ru Y Cao S Gao A . Microrna-101 targets cxcl12-mediated akt and snail signaling pathways to inhibit cellular proliferation and invasion in papillary thyroid carcinoma. Oncol Res. (2019) 27:691–701. doi: 10.3727/096504018X15426763753594 30832753 PMC7848424

[15] Rowe M . An introduction to machine learning for clinicians. Acad Med. (2019) 94:1433–6. doi: 10.1097/ACM.0000000000002792 31094727

[16] Zhang X Jonassen I Goksøyr A . Machine learning approaches for biomarker discovery using gene expression data. In: Nakaya HI, ed. Bioinformatics. Brisbane (AU): Exon Publications (2021). p. 53–64. 33877765

[17] Diaz-Uriarte R Gómez de Lope E Giugno R Fröhlich H Nazarov PV Nepomuceno-Chamorro IA . Ten quick tips for biomarker discovery and validation analyses using machine learning. PloS Comput Biol. (2022) 18:e1010357. doi: 10.1371/journal.pcbi.1010357 35951526 PMC9371329

[18] Alharbi F Vakanski A . Machine learning methods for cancer classification using gene expression data: A review. Bioengineering (Basel). (2023) 10:173. doi: 10.3390/bioengineering10020173 36829667 PMC9952758

[19] Chabon JJ Hamilton EG Kurtz DM Esfahani MS Moding EJ Stehr H . Integrating genomic features for non-invasive early lung cancer detection. Nature. (2020) 580:245–51. doi: 10.1038/s41586-020-2140-0 32269342 PMC8230734

[20] Crosby D Bhatia S Brindle KM Coussens LM Dive C Emberton M . Early detection of cancer. Science. (2022) 375:eaay9040. doi: 10.1126/science.aay9040 35298272

[21] Wei L Niraula D Gates EDH Fu J Luo Y Nyflot MJ . Artificial intelligence (ai) and machine learning (ml) in precision oncology: A review on enhancing discoverability through multiomics integration. Br J Radiol. (2023) 96:20230211. doi: 10.1259/bjr.20230211 37660402 PMC10546458

[22] Mazeh H Deutch T Karas A Bogardus KA Mizrahi I Gur-Wahnon D . Next-generation sequencing identifies a highly accurate mirna panel that distinguishes well-differentiated thyroid cancer from benign thyroid nodules. Cancer Epidemiol Biomarkers Prev. (2018) 27:858–63. doi: 10.1158/1055-9965.EPI-18-0055 30049841

[23] Love MI Huber W Anders S . Moderated estimation of fold change and dispersion for rna-seq data with deseq2. Genome Biol. (2014) 15:550. doi: 10.1186/s13059-014-0550-8 25516281 PMC4302049

[24] Armos R Bojtor B Papp M Illyes I Lengyel B Kiss A . Microrna profiling in papillary thyroid cancer. Int J Mol Sci. (2024) 25:9362. doi: 10.3390/ijms25179362 39273308 PMC11395536

[25] Tian Y Xie T Sun X . Analysis of the regulatory mechanisms of prognostic immune factors in thyroid cancer. Front Oncol. (2022) 12:1059591. doi: 10.3389/fonc.2022.1059591 36591507 PMC9795211

[26] Macerola E Poma AM Vignali P Proietti A Torregrossa L Ugolini C . Microrna expression profiling of ras-mutant thyroid tumors with follicular architecture: Microrna signatures to discriminate benign from malignant lesions. J Endocrinol Invest. (2023) 46:1651–62. doi: 10.1007/s40618-023-02023-5 36749451 PMC10349002

[27] Ren X Zhang J Song Z Li Q Zhang D Li X . Detection of malignant lesions in cytologically indeterminate thyroid nodules using a dual-layer spectral detector ct-clinical nomogram. Front Oncol. (2024) 14:1357419. doi: 10.3389/fonc.2024.1357419 38863637 PMC11165073

[28] Li Y Li T He K Cui X-X Zhang L-L Wei X-L . A predictive nomogram of thyroid nodules based on deep learning ultrasound image analysis. Front Endocrinol. (2025) 16:1504412. doi: 10.3389/fendo.2025.1504412 40365227 PMC12069047

[29] Song Z Liu Q Huang J Zhang D Yu J Zhou B . The value of machine learning based on spectral ct quantitative parameters in the distinguishing benign from malignant thyroid micro-nodules. BMC Cancer. (2025) 25:1041. doi: 10.1186/s12885-025-14450-z 40597056 PMC12211486

[30] Ortiz-Quintero B . Extracellular micrornas as intercellular mediators and noninvasive biomarkers of cancer. Cancers (Basel). (2020) 12:3455. doi: 10.3390/cancers12113455 33233600 PMC7699762

[31] Siddika T Heinemann IU . Bringing micrornas to light: Methods for microrna quantification and visualization in live cells. Front Bioeng Biotechnol. (2021) 8:619583. doi: 10.3389/fbioe.2020.619583 33537295 PMC7848212

[32] Keutgen XM Filicori F Crowley MJ Wang Y Scognamiglio T Hoda R . A panel of four mirnas accurately differentiates malignant from benign indeterminate thyroid lesions on fine needle aspiration. Clin Cancer Res. (2012) 18:2032–8. doi: 10.1158/1078-0432.CCR-11-2487 22351693

[33] Rosenfeld N Aharonov R Meiri E Rosenwald S Spector Y Zepeniuk M . Micrornas accurately identify cancer tissue origin. Nat Biotechnol. (2008) 26:462–9. doi: 10.1038/nbt1392 18362881

[34] Li Y Kowdley KV . Micrornas in common human diseases. Genomics Proteomics Bioinf. (2012) 10:246–53. doi: 10.1016/j.gpb.2012.07.005 23200134 PMC3611977

[35] Sönmez G Ünlütürk U . A systematic review of emerging rna markers in thyroid fine needle aspiration cytology samples: Advancements and challenges. Endocrine. (2025) 89:365–79. doi: 10.1007/s12020-025-04266-z 40346322 PMC12289723

[36] Edsjö A Russnes HG Lehtiö J Tamborero D Hovig E Stenzinger A . High-throughput molecular assays for inclusion in personalised oncology trials - state-of-the-art and beyond. J Intern Med. (2024) 295:785–803. doi: 10.1111/joim.13785 38698538

[37] Liu X Fu Y Zhang G Zhang D Liang N Li F . Mir-424-5p promotes anoikis resistance and lung metastasis by inactivating hippo signaling in thyroid cancer. Mol Ther Oncolytics. (2019) 15:248–60. doi: 10.1016/j.omto.2019.10.008 31890869 PMC6921161

[38] Nwadiugwu MC . Thyroid tumor: Investigating microrna-21 gene suppression in ftc and fta. Cancer Inform. (2020) 19:1176935120948474. doi: 10.1177/1176935120948474 32821081 PMC7412895

[39] Zang C Sun J Liu W Chu C Jiang L Ge R . Mirna-21 promotes cell proliferation and invasion via vhl/pi3k/akt in papillary thyroid carcinoma. Hum Cell. (2019) 32:428–36. doi: 10.1007/s13577-019-00254-4 31161410

[40] Maggisano V Capriglione F Verrienti A Celano M Sponziello M Pecce V . Expression of mir-31-5p affects growth, migration and invasiveness of papillary thyroid cancer cells. Endocrine. (2022) 79:517–26. doi: 10.1007/s12020-022-03267-6 36474133

[41] Zhao H Tang H Huang Q Qiu B Liu X Fan D . Mir-101 targets usp22 to inhibit the tumorigenesis of papillary thyroid carcinoma. Am J Cancer Res. (2016) 6:2575–86. PMC5126274 27904772

[42] Carvalheira G Nozima BH Cerutti JM . Microrna-106b-mediated down-regulation of c1orf24 expression induces apoptosis and suppresses invasion of thyroid cancer. Oncotarget. (2015) 6:28357–70. doi: 10.18632/oncotarget.4947 26317551 PMC4695065

[43] Ma X Wei J Zhang L Deng D Liu L Mei X . Mir-486-5p inhibits cell growth of papillary thyroid carcinoma by targeting fibrillin-1. BioMed Pharmacother. (2016) 80:220–6. doi: 10.1016/j.biopha.2016.03.020 27133060

[44] Chen L Xiong L Hong S Li J Huo Z Li Y . Circulating myeloid-derived suppressor cells facilitate invasion of thyroid cancer cells by repressing mir-486-3p. J Clin Endocrinol Metab. (2020) 105:dgaa344. doi: 10.1210/clinem/dgaa344 32492708

[45] Chen Z Su Y Peng D Wang W Zhong J Zhou A . Circ_0124055 promotes the progression of thyroid cancer cells through the mir-486-3p/mta1 axis. J Endocrinol Invest. (2023) 46:1549–63. doi: 10.1007/s40618-022-01998-x 36604405

[46] Wu H-Y Wei Y Pan S-L . Down-regulation and clinical significance of mir-7-2-3p in papillary thyroid carcinoma with multiple detecting methods. IET Syst Biol. (2019) 13:225–33. doi: 10.1049/iet-syb.2019.0025 31538956 PMC8687168

[47] Qian B Liu Q Wang C Lu S Ke S Yin B . Identification of mir600hg/hsa-mir-342-3p/anln network as a potential prognosis biomarker associated with immune infiltrates in pancreatic cancer. Sci Rep. (2023) 13:15919. doi: 10.1038/s41598-023-43174-y 37741887 PMC10517933

[48] Chen H Wahafu P Wang L Chen X . Lncrna linc00313 knockdown inhibits tumorigenesis and metastasis in human osteosarcoma by upregulating fosl2 through sponging mir-342-3p. Yonsei Med J. (2020) 61:359–70. doi: 10.3349/ymj.2020.61.5.359 32390359 PMC7214116

[49] Yu Y Liao H Xie R Zhang Y Zheng R Chen J . Overexpression of mirna-3613-3p enhances the sensitivity of triple negative breast cancer to cdk4/6 inhibitor palbociclib. Front Oncol. (2020) 10:590813. doi: 10.3389/fonc.2020.590813 33330073 PMC7729088

[50] Tan W Li Z Xia W Zhu J Fan R . Mir-221-3p regulates hepatocellular carcinoma cell proliferation, migration and invasion via targeting lifr. Ann Hepatol. (2021) 27:100567. doi: 10.1016/j.aohep.2021.100567 34699986

[51] Nguyen THN Nguyen TTN Nguyen TTM Nguyen LHM Huynh LH Phan HN . Panels of circulating micrornas as potential diagnostic biomarkers for breast cancer: a systematic review and meta-analysis. Breast Cancer Res Treat. (2022) 196:1–15. doi: 10.1007/s10549-022-06728-8 36085533

[52] Di Martino MT Arbitrio M Caracciolo D Cordua A Cuomo O Grillone K . Mir-221/222 as biomarkers and targets for therapeutic intervention on cancer and other diseases: a systematic review. Mol Ther Nucleic Acids. (2022) 27:1191–224. doi: 10.1016/j.omtn.2022.02.005 35282417 PMC8891816

[53] Lukyanov SA Sergiyko SV Titov SE Beltsevich DG Veryaskina YA Vanushko VE . New opportunities for preoperative diagnosis of medullary thyroid carcinoma. Biomedicines. (2023) 11:1473. doi: 10.3390/biomedicines11051473 37239145 PMC10216661

[54] Dowell N Begum S Muzaffar J Boelaert K Nieto H . Comparing the diagnostic accuracy of afirma gsc to thyroseq v3 in cytologically indeterminate thyroid nodules. Eur Thyroid J. (2025) 14:e250296. doi: 10.1530/etj-25-0296 41324437 PMC12697243

[55] Vardarli I Tan S Görges R Krämer BK Herrmann K Brochhausen C . Diagnostic accuracy of afirma gene expression classifier, afirma gene sequencing classifier, thyroseq v2 and thyroseq v3 for indeterminate (bethesda iii and iv) thyroid nodules: a meta-analysis. Endocr Connect. (2024) 13:e240170. doi: 10.1530/EC-24-0170 38771544 PMC11227067

[56] Zhong L Shi L Lai J Hu Y Gu L . Combined model integrating clinical, radiomics, braf(v600e) and ultrasound for differentiating between benign and malignant indeterminate cytology (bethesda iii) thyroid nodules: a bi-center retrospective study. Gland Surg. (2024) 13:1954–64. doi: 10.21037/gs-24-310 39678423 PMC11635559

[57] Wu L Shu H Chen W Gao Y Yuan Y Li X . Diagnostic value of thyroid imaging reporting and data system combined with brafv600e mutation analysis in bethesda categories iii-v thyroid nodules. Sci Rep. (2022) 12:5934. doi: 10.1038/s41598-022-09822-5 35395862 PMC8993851

