# Supplementary material for: Integrating miRNA profiling and machine learning for improved thyroid cancer diagnosis
Source: Front Oncol. 2026 Sep 7;16:1838366. doi: 10.3389/fonc.2026.1838366 (PMC13591501; doi:10.3389/fonc.2026.1838366)
Supplement: Supplementary file 1 [file Table1.docx]

Supplementary Table 1. Evaluation metrics for these 5 machine learning algorithms.

|  | Model | AUC | Accuracy | Kappa | Sensitivity | Specificity | Precision | F1_Score | Balanced_Accuracy |
| --- | --- | --- | --- | --- | --- | --- | --- | --- | --- |
| 1 | GLM | 0.774575 | 0.64486 | 0.241701 | 0.512195 | 0.727273 | 0.538462 | 0.525 | 0.619733925 |
| 2 | RF | 0.699002 | 0.672897 | 0.26381 | 0.414634 | 0.833333 | 0.607143 | 0.492754 | 0.62398374 |
| 3 | SVM | 0.787879 | 0.672897 | 0.298295 | 0.536585 | 0.757576 | 0.578947 | 0.556962 | 0.647080562 |
| 4 | XGBoost | 0.695492 | 0.691589 | 0.31926 | 0.487805 | 0.818182 | 0.625 | 0.547945 | 0.652993348 |
| 5 | KNN | 0.780673 | 0.71028 | 0.372612 | 0.560976 | 0.80303 | 0.638889 | 0.597403 | 0.682002956 |
